# An assessment of the quality of current clinical meta-analyses

**DOI:** 10.1186/s12874-020-00999-9

**Published:** 2020-05-07

**Authors:** Irbaz Hameed, Michelle Demetres, Derrick Y. Tam, Mohamed Rahouma, Faiza M. Khan, Drew N. Wright, Keith Mages, Antonio P. DeRosa, Becky Baltich Nelson, Kevin Pain, Diana Delgado, Leonard N. Girardi, Stephen E. Fremes, Mario Gaudino

**Affiliations:** 1 Department of Cardiothoracic Surgery, Weill Cornell Medicine, 525 East 68th Street, New York, NY 10065 USA; 2 Samuel J. Wood Library and C.V. Starr Biomedical Information Centre, Weill Cornell Medicine, New York, NY USA; 3 Schulich Heart Centre, Sunnybrook Health Sciences Centre, University of Toronto, Toronto, Canada

**Keywords:** Meta-analysis, PRESS, PRISMA, IOM, Cochrane, Clinical, Quality, Methodology, Epidemiology

## Abstract

**Background:**

The objective of this study was to assess the overall quality of study-level meta-analyses in high-ranking journals using commonly employed guidelines and standards for systematic reviews and meta-analyses.

**Methods:**

100 randomly selected study-level meta-analyses published in ten highest-ranking clinical journals in 2016–2017 were evaluated by medical librarians against 4 assessments using a scale of 0–100: the Peer Review of Electronic Search Strategies (PRESS), Preferred Reporting Items for Systematic Reviews and Meta-Analyses (PRISMA), Institute of Medicine’s (IOM) Standards for Systematic Reviews, and quality items from the Cochrane Handbook. Multiple regression was performed to assess meta-analyses characteristics’ associated with quality scores.

**Results:**

The overall median (interquartile range) scores were: PRESS 62.5(45.8–75.0), PRISMA 92.6(88.9–96.3), IOM 81.3(76.6–85.9), and Cochrane 66.7(50.0–83.3). Involvement of librarians was associated with higher PRESS and IOM scores on multiple regression. Compliance with journal guidelines was associated with higher PRISMA and IOM scores.

**Conclusion:**

This study raises concerns regarding the reporting and methodological quality of published MAs in high impact journals Early involvement of information specialists, stipulation of detailed author guidelines, and strict adherence to them may improve quality of published meta-analyses.

## Background

In the pyramid of evidence-based medicine, systematic reviews and meta-analyses (MAs) are considered the highest level and have become the preferred source for professional guidelines and recommendations [1]. In recent years, the scientific community has witnessed an almost exponential increase in the annual number of MAs published and it has been estimated that currently eleven new MAs are published every day [2].

Study level MAs are often seen as an attractive first step to publication for young investigators with limited resources as they do not require ethics approval, primary data collection, and can be performed relatively quickly given the availability of data. Furthermore, the review process for MAs may be less effective than for traditional original studies due to the lack of familiarity of editors and reviewers with the meta-analytic approaches [3]. This has led to controversy surrounding the quality of published MAs on several occasions in terms of methodology and reproducibility [4, 5]. Given the importance placed on MA in the synthesis of evidence and its use in clinical decision making, understanding factors affecting the quality of a published MA is of paramount importance.

Previously, studies evaluating the quality of the specific components of MAs have been published. However, these investigations focused mainly on the search strategy or on the adherence to reporting guidelines like the Preferred Reporting Items for Systematic Reviews and Meta-Analyses (PRISMA). To date, a comprehensive evaluation of the methodological quality of the published meta-analytic evidence has not been reported.

In this study we performed a comprehensive quality assessment of recently published clinical MAs from high impact journals using commonly employed systematic review and MA reporting guidelines and standards.

## Methods

### Journal and meta-analysis selection

The study protocol was defined a priori and there were no deviations from the protocol (Supplementary appendix). The highest-ranking clinical journals publishing study level MAs were identified by a search performed on SCImago [6] on August 24th, 2018. Of these, the 10 highest ranking journals publishing ≥10 study level MAs between January 2016 and December 2017 were included. For journals belonging to the same family, the highest impact journal was selected. As MAs can be performed with any two or more independent studies, we assessed only systematic review-derived MAs, i.e. MAs that were the result of a systematic review literature search. The systematic review-derived MAs published in these journals were extracted from PubMed by a medical librarian with the following query:

“Journal name”[Journal] AND (“meta-analysis”[Publication Type] OR “meta-analysis as topic”[MeSH Terms] OR “meta-analysis”[All Fields]) AND (“2016/01/01”[PDAT]: “2017/12/31”[PDAT]).

The search results were independently screened by two reviewers to select study level MAs only and exclude patient-level MAs. Discrepancies on inclusion were resolved by consensus. A number was assigned to each selected study level MA and 100 MAs were selected through a computer-based random number generation for analysis (R (version 3.3.3 R Project for Statistical Computing) within RStudio (0.99.489, http://www.rstudio.com)).

For each journal, the following data were extracted: SCImago Journal ranking, 2017 journal impact factor (Clarivate Journal Citation Reports-Thomson Reuters), and availability of detailed journal author guidelines for MAs. For each of the 100 MAs selected, the following variables were extracted: journal name, year of publication, clinical field, involvement of librarian in systematic review, databases searched, reporting tool cited, compliance with journal author guidelines, MA of randomized controlled trials (RCTs) only, network MA, and number of studies included in the MA.

### Quality assessment and scoring

Seven systematic review medical librarians assessed the MAs. Each librarian is specially trained on the methodology/conduct of systematic reviews/MAs with published work in the field. For each MA, two librarians independently extracted and assessed each data item on each of the four guidelines/standards.

Articles were assessed using the following systematic review guidelines and quality standards: (1) the Peer Review of Electronic Search Strategies (PRESS) checklist; (2) the PRISMA checklist; (3) the Institute of Medicine’s (IOM) Standards for Systematic Reviews; and (4) quality items from the Cochrane Handbook of Systematic Reviews. This four-tiered evaluation was aimed at assessing not only the level of data reporting (PRISMA), but also the quality of the systematic search (PRESS, Cochrane) and of the overall MA (IOM). Scoring was developed by the authors.

The PRESS checklist [7], designed to evaluate a systematic review search strategy, is divided into six categories: translation of the research question; Boolean and proximity operators; subject headings; text word searching; spelling, syntax and line numbers; and limits and filters. Due to the subjective nature of a systematic review search, two librarians independently evaluated and scored the MAs against each PRESS checklist category as follows: 0 – not applicable, 1 – not addressed; 2 - mostly incomplete; 3 - mostly complete; 4 – fully addressed and inter-observer agreement between librarians was determined. Thus, the maximum score per applicable item was 4, and the maximum total possible score for a MA was 4 times the number of applicable items for that MA (a MA with all items applicable can score a maximum of 24). If a MA scored “not applicable” in all categories, it was excluded from the analysis for PRESS score. The lower of the two scores from the librarians was used as the final score for an MA.

PRISMA [8], is a list of reporting items that should appear in a systematic review/MA. However, it does not speak to the quality of, or degree to which these items have been reported. Consequently, for PRISMA items, MAs were scored as follows: 0 – not reported; 1 – reported. The maximum possible score for each MA was 27 (the total number of checklist items). Discrepancies were discussed among the librarians to achieve consensus.

IOM’s Standards for Systematic Reviews (standards 2.1 through 5.1) [9] assess the complete body of a systematic review/MA, addressing the quality of the MA from inception (where possible) to final reporting. The subcategories under each IOM standard were scored as follows: 0 - not applicable; 1 – not addressed; 2 – mostly incomplete; 3 - mostly complete; 4 – fully addressed. The maximum score per applicable item was 4 and hence the maximum total possible score for a MA was 4 times the number of its applicable items (a MA with all items applicable can score a maximum of 64). Discrepancies were discussed among the librarians to achieve consensus.

Quality items identified by the Cochrane Handbook of Systematic Reviews [10] as important to ensure reproducibility of MA searches were also abstracted. These items were pulled as they were not fully addressed by the other evaluation tools and are vital to the reproducibility of the searches. These were reporting (i) the date of the search; (ii) the number of databases searched; (iii) the full search strategy for all databases included; (iv) the platform used to search each database; (v) claiming a reporting tool; and (vi) appropriateness of the reporting tool. The scoring was as follows: 0 – not reported; 1 – reported. The maximum possible score for each MA was 6 (the number of quality items included). Discrepancies were discussed among the librarians to achieve consensus.

As a secondary analysis, the four quality assessment scores of each MA were then converted into a score from 0 to 100 by dividing the individual score by the maximum possible score for that assessment and multiplying by 100. An overall quality score was calculated for each MA as the mean of the four individual quality assessment scores (Supplementary Table 1). If a MA scored “not applicable” in all categories of the PRESS score (ie. did not provide a search strategy), the PRESS score was not included in the calculation of the overall quality score, which in this situation was determined as the average of the other 3 quality assessments.

### Statistical analysis

The Wilks-Shapiro test was used to assess normality for the final checklist scores. For each checklist, the mean and standard deviation (SD) or the median and the inter-quartile ranges (IQR) were calculated for normally and non-normally distributed scores respectively. Descriptive bivariate analysis was used to compare each score against binary variables using the Student’s T-test for normally distributed scores and Mann Whitney U test for non-normally distributed scores. For categorical variables with more than 2 levels, analysis of variance (ANOVA) and Kruskal Wallis Test were used for normally and non-normally distributed scores, respectively. Correlation between continuous variables and scores were also calculated and reported as Pearson’s correlation coefficient (r).

For PRESS checklist, Cohen’s Kappa value for inter-observer agreement between librarians was determined due to its subjective evaluation. The Kappa result was interpreted as follows: values ≤0 as indicating no agreement and 0.01–0.20 as none to slight, 0.21–0.40 as fair, 0.41–0.60 as moderate, 0.61–0.80 as substantial, and 0.81–1.00 as almost perfect agreement [11].

Multiple linear regression were conducted on the following variables to identify factors associated with each of the standard checklists (PRESS, PRISMA, and IOM) as well as the overall score: librarian Involvement, clinical field, compliance with journal guidelines, impact factor (Clarivate Analytics), MA of RCT only, network MA, and number of included studies.

The clinical field associated with highest mean scores served as the reference in the regression models.

*P* value < 0.05 was considered statistically significant and 95% confidence intervals were calculated. Analyses were performed using R (version 3.3.3 R Project for Statistical Computing) within RStudio (0.99.489, http://www.rstudio.com).

## Results

### Journal and meta-analyses selection

The top 25 clinical journals reporting MAs (patient-level and study level) from 2016 to 2017 are shown in Supplementary Table 2. Of these, the 10 highest ranking journals reporting ≥10 study level MAs were: Lancet, Journal of American College of Cardiology, European Heart Journal, Circulation, the Journal of the American Medical Association, Gastroenterology, Annals of the Rheumatic Diseases, Annals of Internal Medicine, Gut, and World Psychiatry (Supplementary Table 3).

A total of 302 systematic reviews and MAs published in these journals were retrieved, from which 148 study level MAs were identified. 100 of the 148 MAs were selected randomly and formed our sample; there were 72 MAs of observational studies and RCTs, 28 MAs of RCTs only. Ten of the MAs were network MAs (Table 1). The number of MAs selected from each journal are shown in Supplementary Table 3. These MAs were categorized under the following broad clinical fields: cardiovascular, gastroenterology, preventive medicine and epidemiology, psychiatry, and rheumatology (Table 1).

**Table 1:** Details of included meta-analyses

Variable	Number
Number of Meta-Analyses	100 (100.0)
Librarian Involved (%)	20 (20.0)
Journal (%)	
➢ Annals of Internal Medicine	12 (12.0)
➢ Annals of The Rheumatic Diseases	10 (10.0)
➢ Circulation	10 (10.0)
➢ European Heart Journal	13 (13.0)
➢ Gastroenterology	12 (12.0)
➢ Gut	8 (8.0)
➢ Journal of American College of Cardiology	11 (11.0)
➢ The Journal of The American Medical Association	10 (10.0)
➢ Lancet	7 (7.0)
➢ World Psychiatry	7 (7.0)
Journal Impact Factor (2017 Clarivate) (Mean (SD))	24.79 (12.2)
ScImago Rank (Mean (SD))	8.96 (2.0)
Journal Guidelines Availability (%)	39 (39.0)
Compliance with Journal Guidelines (%)	39 (39.0)
Area (%)	
➢ Cardiovascular	40 (40.0)
➢ Gastroenterology	24 (24.0)
➢ Preventive Medicine and Epidemiology	15 (15.0)
➢ Psychiatry	9 (9.0)
➢ Rheumatology	12 (12.0)
Meta-analysis of randomized controlled trials (%)	28 (28.0)
Network meta-analysis (%)	10 (10.0)
Number of Included Studies meta-analysis (Mean (SD))	45.0 (57.5)

Librarians were involved in 20 MAs. 78 MAs reported searching 3 or more databases. 37 MAs reported detailed search strategies for each database included. (Table 2) The most frequently searched databases were EMBASE, MEDLINE and PubMed (Table 3).

**Table 2:** Additional quality assessments from the Cochrane Handbook scoring

Quality assessment	Number of meta-analyses reporting item (%)
Date of search provided	88 (88)
Number of databases searched ≥3	78 (78)
*Breakdown of number of databases searched:*	
➢ Not reported	2 (2)
➢ 1	6 (6)
➢ 2	14 (14)
➢ 3	42 (42)
➢ 4	12 (12)
➢ 5	10 (10)
➢ 6	5 (5)
➢ 7	4 (4)
➢ 8	3 (3)
➢ 9	1 (1)
➢ 14	1 (1)
Included searches for all databases^a^	37 (37)
Included platform for each database^b^	23 (23)
Claimed a reporting tool	72 (72)
Is the reporting tool appropriate^c^	71 (71)

^a^Not applicable for 1 meta-analysis

^b^Not applicable for 2 meta-analyses

^c^Not applicable for 28 meta-analyses

**Table 3:** Databases searched in the randomly selected 100 meta-analyses (in decreasing frequency)

Database searched	Number of Papers (%)
EMBASE	67 (67)
MEDLINE	59 (59)
PubMed	54 (54)
Cochrane Library	44 (44)
Web of Science	22 (22)
SCOPUS	16 (16)
CINAHL	11 (11)
PsycINFO	10 (10)
Clinicaltrials.gov	10 (10)
LILACS	3 (3)
AMED	2 (2)
BIOSIS	2 (2)
EBSCO	1 (1)
TOXNET	1 (1)
Google Scholar	1 (1)
HMIC	1 (1)

### Quality assessment

**Fig. 1:**
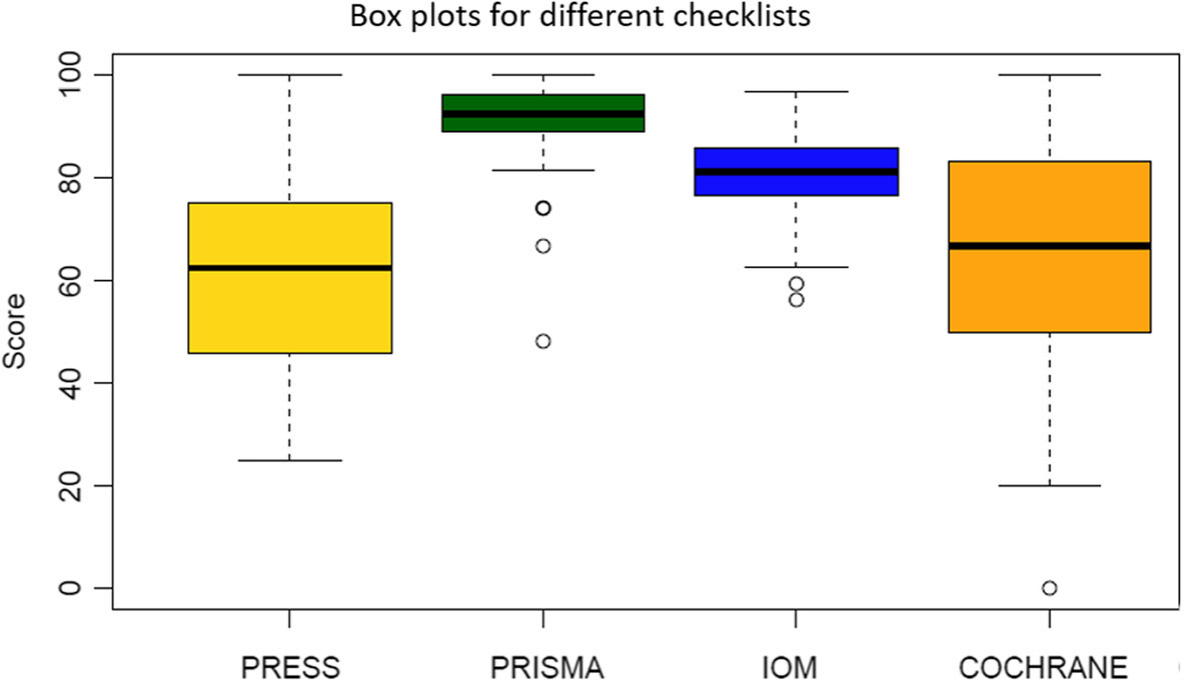
Box plots for scores obtained by meta-analyses under different scoring items. Interpretation: upper horizontal line of box, 75th percentile; lower horizontal line of box, 25th percentile; thick horizontal bar within box, median; upper horizontal bar outside box, 90th percentile; lower horizontal bar outside box, 10th percentile. Circles represent outliers

The summaries for the scoring of each checklist are shown in Fig. 1 and Supplementary Table 1 respectively. The mean (SD) PRESS score was 61.6 (19.1). 5 of the MAs were graded as “not applicable” for all PRESS assessments. The median (IQR) PRISMA score was 92.6 (88.9–96.3). Four journals stipulated adherence to PRISMA guidelines and in total, 67 MAs were guided by PRISMA. The mean (SD) IOM score was 81.0 (8.2), and the median (IQR) Cochrane score was 66.7 (50.0–83.3).

The mean (SD) Global quality score was 74.4 (8.5). Detailed scoring for each item on the checklists are presented in Table 2 and Supplementary Tables 4–6.

### Inter-rater reliability

Inter-observer agreement for PRESS checklist was fair for “text word searching” (Kappa 0.30), and moderate for “translation of research question”, “Boolean and proximity operators”, “subject headings”, “spelling, syntax and line numbers”, and “limits and filters” (Kappa 0.55, 0.49, 0.55, 0.54, and 0.43, respectively) (Supplementary Table 7).

### Factors associated with quality scores

Involvement of librarians was associated with significantly higher PRESS scores (72.3 [19.0] vs non-involvement of librarians, 58.9 [18.2], *p* < 0.01). Availability of author guidelines for MA and author compliance was associated with significantly higher PRISMA scores (96.3, 92.6–100.0, vs non-availability, 92.6, 88.9–96.3, p < 0.01) and IOM scores (85.0 [7.2) vs non-availability 78.4 [7.8], *p* < 0.001). Network MAs were associated with significantly higher PRISMA score only (98.2, 92.6–100.0) vs other MAs (92.6, 88.9–96.3, *p* = 0.04). No significant association was observed between the checklist scores, and clinical fields or MAs of RCTs (Table 4). There was no correlation between the number of studies included in the MAs, the journal impact factors (2017 Clarivate), and the ScImago ranking of journals and the checklist scores (Supplementary Table 8).

**Table 4:** Association of variables with individual checklist scores

	Number	PRESS Score (mean ± SD)	PRISMA Score (median (IQR))	IOM Score (mean ± SD)
LIBRARIAN INVOLVEMENT				
Yes	20	72.3 (19.0)	92.6 [91.7, 96.3]	83.1 (6.4)
No	80	58.9 (18.2)	92.6 [88.9, 96.3]	80.4 (8.5)
*P*-value	–	**< 0.01**	0.673	0.186
CLINICAL FIELD				
Cardiovascular	40	56.5 (20.3)	92.6 [88.9, 96.3]	80.1 (8.6)
Gastroenterology	24	60.3 (19.1)	96.3 [92.6, 96.3]	79.8 (8.2)
Preventive medicine and epidemiology	15	66.2 (13.2)	96.30 [88.89, 98.15]	84.48 (9.59)
Psychiatry	9	63.5 (19.8)	92.6 [88.9, 96.3]	80.6 (6.2)
Rheumatology	12	71.7 (18.2)	92.6 [88.0, 96.3]	82.0 (5.7)
P-value	–	0.14	0.49	0.42
COMPLIANCE WITH JOURNAL GUIDELINES				
Guidelines available and full compliance	39	59.8 (16.9)	96.3 [92.6, 100.0]	85.0 (7.2)
Guidelines unavailable	61	62.8 (20.4)	92.6 [88.9, 96.3]	78.4 (7.8)
P-value	–	0.46	**< 0.01**	**< 0.001**
MA of RCTs ONLY				
Yes	28	57.2 (17.9)	96.3 [91.7, 100.0]	83.0 (8.2)
No	72	63.1 (19.4)	92.6 [88.9, 96.3]	80.1 (8.1)
P-value	–	0.19	0.10	0.11
NETWORK MA				
Yes	10	55.0 (18.8)	98.2 [92.6, 100.0]	83.6 (7.5)
No	90	62.3 (19.1)	92.6 [88.9, 96.3]	80.7 (8.3)
*P*-value	–	0.28	**0.04**	0.29

*IOM* Institute of Medicine, *IQR* interquartile range, *MA* Meta-analysis, *PRESS* Peer Review of Electronic Search Strategies, *PRISMA* Preferred Reporting Items for Systematic Reviews and Meta-Analyses, *RCT* randomized controlled trial, *SD* standard deviation

Librarian involvement was also associated with higher PRESS scores (Beta = 12.56, 95% CI 2.87–22.25, *p* < 0.05) and IOM scores (Beta = 3.99, 95% CI 0.13–7.85, *p* < 0.05) but not PRISMA score (Beta = 3.58, 95% CI -0.66 – 7.82, *p* < 0.1). Compliance with journal guidelines was associated with higher PRISMA (Beta = 3.07, 95% CI 0.82–5.32, *p* < 0.01) and IOM scores (Beta = 4.34, 95% CI 2.29–6.38), p < 0.01). (Table 5).

**Table 5:** Predictors of PRESS, PRISMA, and IOM scores

	***Dependent variable***		
	PRESS	PRISMA	IOM
Librarian Involvement	12.56^**^	3.58	3.99^**^
	**(2.87, 22.25)**	(−0.66, 7.82)	**(0.13, 7.85)**
Field - Preventive Medicine and Epidemiology	−1.54	*Reference*	*Reference*
	(−18.10, 15.03)		
Field - Cardiovascular	−11.28^*^	0.42	−0.84
	(−24.10, 1.55)	(−5.46, 6.29)	(−6.18, 4.51)
Field - Gastroenterology	−10.03	1.99	0.67
	(−22.85, 2.79)	(−4.48, 8.47)	(−5.21, 6.56)
Field - Psychiatry	−1.81	2.42	2.45
	(−18.80, 15.18)	(−5.38, 10.22)	(−4.64, 9.55)
Field - Rheumatology	*Reference*	1.49 (−5.89, 8.87)	2.21
			(−4.50, 8.92)
Compliance with journal guidelines	−0.40	3.07^***^	4.34^***^
	(−5.58, 4.77)	**(0.82, 5.32)**	**(2.29, 6.38)**
Journal Impact Factor (2017 Clarivate)	−0.15	− 0.06	− 0.10
	(− 0.54, 0.23)	(− 0.23, 0.12)	(− 0.26, 0.05)
Meta-analysis of randomized controlled trials	−3.01	0.73	1.95
	(−12.75, 6.73)	(−3.46, 4.92)	(−1.86, 5.76)
Network meta-analysis	−3.24	4.72	2.36
	(−17.34, 10.86)	(−1.32, 10.77)	(− 3.13, 7.86)
Number of included studies	0.02	−0.002	0.002
	(−0.05, 0.09)	(−0.03, 0.03)	(− 0.03, 0.03)
Constant	71.23^***^	82.03^***^	69.49^***^
	(55.00, 87.47)	(72.23, 91.83)	(60.58, 78.40)
Observations	95	100	100
R^2^	0.17	0.13	0.24
Adjusted R^2^	0.07	0.04	0.16
Residual Std. Error (df = 89)	18.35 (df = 84)	8.26	7.52
F Statistic (df = 10; 89)	1.74^*^ (df = 10; 84)	1.39	2.86^***^

*IOM* Institute of Medicine, *PRESS* Peer Review of Electronic Search Strategies, *PRISMA* Preferred Reporting Items for Systematic Reviews and Meta-Analyses

^*^p < 0.1; ^**^p < 0.05; ^***^p < 0.01,

## Discussion

The exponential increase in the number of MAs published since the late 1990s have raised concerns on the quality of conduct of these MAs. While previous studies examining quality of MAs focused on individual aspects of quality, we comprehensively examined the quality in a random sample of 100 study-level MAs published in ten high ranking clinical journals. We found that the majority of these MAs had poorer performances for PRESS and Cochrane assessments, and relatively stronger performance in PRISMA and IOM.

The strongest predictor of the higher PRESS and IOM scores was librarian involvement. This suggests that the key to improving the overall quality of MAs is to include a trained information specialist – likely driven by the fact that at least three checklist scores have a component related to the search strategy of the study. This finding is reflected in current literature. In an analysis of systematic reviews using PRESS and IOM checklists, Rethlefsen et al. found the level of librarian and information specialist participation to be significantly associated with search reproducibility from reported search strategies [12]. Koffel surveyed 1560 authors of systematic reviews and reported librarian involvement to be significantly associated with the use of recommended search methods [13]. In practice, not all researchers performing MAs are able to collaborate with systematic review trained information specialists, and in our analysis, only 20% of the assessed MAs reported librarian or information specialist participation. However, if journals guidelines for MA encourage inclusion of information specialists as collaborators, their important role can be emphasized, and more resources made available by institutions. Of note, none of the journals included in our analysis mandated the inclusion of a librarian or information specialist in the process. A reproducible and comprehensive systematic literature search is the cornerstone to a rigorous MA and the exclusion of relevant studies due to a poor systematic review search will bias the findings of the analysis. These findings suggest again that journals need to encourage the use of trained personnel to conduct thorough systematic reviews of the literature and require the submission of a complete literature search strategy for the review process to ensure reproducibility and transparency of the search.

Notably, journal ranking and impact factor had no impact on the overall quality of the MA suggesting that higher impact journals were not more rigorous than relatively lower impact journals in assessing MAs. While this finding is echoed in some studies, it is in opposition to others. Ruano et al. recently evaluated quality of systematic reviews and MAs on psoriasis and found no association between quality and bibliometric factors [14]. However, Fleming et al. reported in 2014 that systematic reviews published in higher impact journals were undertaken more rigorously (per impact factor unit: β = 0.68%; 95% CI 0.32–1.04; *P* < 0.001) [15]. Instead, there was a non-significant trend to suggest that the cardiovascular clinical field may be associated with low quality in reporting search strategy (PRESS). It is important to note that two out of three of the cardiovascular journals had no requirements for adherence to reporting or conduct guidelines, suggesting the importance of providing reviewers with standards and checklists in the evaluation of MAs.

Only four of the ten highest ranking journals publishing study level MAs, required investigators to follow reporting guidelines. Not surprisingly, high PRISMA scores, which represent good reporting practices, were strongly associated with availability of author guidelines for the conduct of MAs. Thus, to improve the overall reporting of MAs, journals should mandate authors to follow a standardized checklist to ensure that all aspects of the MA are properly reported to ensure MA reproducibility and reduce bias in outcome reporting. Similarly, compliance with guidelines and the availability of MA guidelines were strong predictors of IOM score, which assesses the quality of the MA from inception to final publication. The review process should also consider using checklists and guidelines to evaluate the submission to ensure a standardized approach to assessing quality.

### Limitations

The above findings must be interpreted in the context of some important limitations. First, we included a random sample of 100 MAs from the ten highest ranking medical journals and this may limit generalizability of findings to lower impact journals. However, we selected high ranking journals to show that even high-ranking journals which may be perceived to be of the highest quality and rigor were not immune to methodological shortcomings. Importantly, we showed in our analysis that impact factor was not a predictor of quality. Furthermore, our sample size of 100 may be underpowered to detect differences in some outcomes. We recognize that our summary overall quality score has not been previously validated. In addition, we recognize that some of the tools (PRISMA, IOM) are subjective and were not designed with the intention of generating scores. The interpretations of these checklists should be evaluated taking this into consideration. We evaluated the methodological rigor and reproducibility of the published meta-analyses in the context of the most commonly referenced guidelines and checklists. The AMSTAR 2 checklist was published in September, 2017 as a critical appraisal tool for MAs [16]. Since the tool is not typically referenced in the performance and writing of systematic reviews/meta analyses and was published after majority of our included MAs, it was not used to assess the methodological quality and reproducibility of these studies. The use of a total score for each individual quality assessment as a continuous scale suggests that each incremental point results in higher or lower quality and may not capture the true impact of violation/infraction on MA quality. In our analysis, we converted a series of 4-point Likert scale questions to a continuous score. We acknowledge that severely flawed MA may still achieve a high score in any checklist, and thus, summarizing scores as a continuous variable may potentially under-report truly flawed studies. Finally, the thresholds for what would be considered a “good” or “bad” study with our overall score are not known. Instead, we assume that a higher numerical score equated to better overall MA.

## Conclusion

Our study raises concerns regarding the reporting and methodological quality of published MAs in high impact journals. Early involvement of information specialists, stipulation of detailed author guidelines, and strict adherence to them may improve quality of published MAs.

## Supplementary information

**Additional file 1: Table S1.** Summary of quality assessments and scoring Study Protocol. **Table S2.** Top tier clinical journals (SCImago ranking) reporting meta-analyses. **Table S3.** The highest-ranking clinical journals (SCImago) reporting aggregate-level meta-analyses and the number of included meta-analyses in our quality assessment. **Table S4.** Peer Review of Electronic Search Strategies (PRESS) checklist scoring by two librarians. For the points in each section of the PRESS checklist, if none or less than half were addressed, it was scored as “not addressed” or “mostly incomplete.” If half, more than half, or all were addressed, it was scored as “mostly complete” or “fully addressed.” “Not applicable” was used if the point was not relevant to the instance or unable to be evaluated. **Table S5.** Preferred Reporting Items for Systematic Reviews and Meta-Analyses (PRISMA) Checklist scoring. **Table S6.** Institute of Medicine (IOM) checklist scoring. For the points in each section of the IOM checklist, if none or less than half were addressed, it was scored as “not addressed” or “mostly incomplete.” If half, more than half, or all were addressed, it was scored as “mostly complete” or “fully addressed.” “Not applicable” was used if the point was not relevant to the instance or unable to be evaluated. **Table S7.** Inter-observer agreement between librarians for Peer Review of Electronic Search Strategies (PRESS) checklist. **Table S8.** Correlation (r) between continuous meta-analyses variables and checklist scores.

**Additional file 2.** Study protocol.

## Data Availability

The datasets used and/or analysed during the current study are available from the corresponding author on reasonable request.

## Abbreviations

ANOVA: Analysis of variance
IOM: Institute of Medicine
IQR: Inter-quartile range
MA: meta-analysis
PRESS: Peer Review of Electronic Search Strategies
PRISMA: Preferred Reporting Items for Systematic Reviews and Meta-Analyses
RCT: Randomized controlled trial
SD: Standard deviation
