# Supplementary material for: An assessment of the quality of current clinical meta-analyses
Source: BMC Med Res Methodol. 2020 May 7;20:105. doi: 10.1186/s12874-020-00999-9 (PMC7204021; doi:10.1186/s12874-020-00999-9)
Supplement: Supplementary file 2 — Additional file 2. Study protocol. [file 12874_2020_999_MOESM2_ESM.docx]

**STUDY PROTOCOL**

**An assessment of the quality of current clinical meta-analyses**

**MATERIALS AND METHODS**

***Journal and meta-analysis selection***

The highest-ranking clinical journals publishing study level MAs will be identified using a standardized journal ranking platform. Of these, the 10 highest ranking journals publishing at least 10 study level MAs between January 2016 and December 2017 will be included. The cut-off of 10 studies is defined as some high impact journals have policies of not publishing study-level MAs and other journals publish only individual patient data meta-analyses.

For multiple journals belonging to the same family, the highest impact journal will be selected. The systematic review-derived MAs published in these journals will be extracted from PubMed by a medical librarian using a detailed search.

The search results will be independently screened by two reviewers to select study level MAs only and exclude patient-level MAs. Discrepancies on inclusion will be resolved by consensus. A number will be assigned to each selected study level MA and 100 MAs will be selected through a computer-based random number generation for analysis.

For each journal, the following data will be extracted: SCImago Journal ranking, 2017 journal impact factor (Clarivate Journal Citation Reports -Thompson Reuters), and availability of detailed journal author guidelines for meta-analysis. For each of the 100 MAs selected, the following variables will be extracted: journal name, year of publication, clinical field, involvement of librarian in systematic review, databases searched, reporting tool cited, compliance with journal author guidelines, MA of randomized controlled trials (RCTs) only, network MA, and number of studies included in the MA.

***Quality assessment and scoring***

Our institution’s librarians will assess each selected MAs. All librarians are specially trained on the methodology/conduct of systematic reviews/meta analyses with published work in the field. For each MA, two librarians shall independently extract and assessed each data item on each of the four guideline/standards.

Articles shall be assessed using the following systematic review guidelines and quality standards: (1) the Peer Review of Electronic Search Strategies (PRESS) checklist; (2) the PRISMA checklist; (3) the Institute of Medicine's (IOM) Standards for Systematic Reviews; and (4) quality items from the Cochrane Handbook of Systematic Reviews. This four-tiered evaluation is aimed at assessing not only the level of data reporting (PRISMA), but also the quality of the systematic search (PRESS, Cochrane) and of the overall MA (IOM). Scoring was developed by the authors.

The PRESS checklist, designed to evaluate a systematic review search strategy, is divided into six categories: translation of the research question; Boolean and proximity operators; subject headings; text word searching; spelling, syntax and line numbers; and limits and filters. Due to the subjective nature of a systematic review search, two librarians shall independently evaluate and score the MAs against each PRESS checklist category as follows: 0 – not applicable, 1 – not addressed; 2 - mostly incomplete; 3 - mostly complete; 4 – fully addressed and inter-observer agreement between librarians was determined. Thus, the maximum score per applicable item shall be 4, and the maximum total possible score for a MA will be 4 times the number of applicable items for that MA (a MA with all items applicable can score a maximum of 24). If a MA scores “not applicable” in all categories, it will be excluded from the analysis for PRESS score. The lower of the two scores from the librarians shall be used as the final score for an MA.

PRISMA, is a list of reporting items that should appear in a systematic review/MA. However, it does not speak to the quality of, or degree to which these items have been reported. Consequently, for PRISMA items, MAs will be scored as follows: 0 – not reported; 1 – reported. The maximum possible score for each MA is 27 (the total number of checklist items). Discrepancies will be discussed among the librarians to achieve consensus.

IOM's Standards for Systematic Reviews (standards 2.1 through 5.1) assess the complete body of a systematic review/MA, addressing the quality of the MA from inception (where possible) to final reporting. The subcategories under each IOM standard will be scored as follows: 0 - not applicable; 1 – not addressed; 2 – mostly incomplete; 3 - mostly complete; 4 – fully addressed. The maximum score per applicable item is 4 and hence the maximum total possible score for a MA will be 4 times the number of its applicable items (a MA with all items applicable can score a maximum of 64). Discrepancies will be discussed among the librarians to achieve consensus.

Quality items identified by the Cochrane Handbook of Systematic Reviews as important to ensure reproducibility of MA searches shall also be abstracted. These items will be pulled as they are not fully addressed by the other evaluation tools and are vital to the reproducibility of the searches. These include reporting (i) the date of the search; (ii) the number of databases searched; (iii) the full search strategy for all databases included; (iv) the platform used to search each database; (v) claiming a reporting tool; and (vi) appropriateness of the reporting tool. The scoring will be as follows: 0 – not reported; 1 – reported. The maximum possible score for each MA will be 6 (the number of quality items included). Discrepancies shall be discussed among the librarians to achieve consensus.

As a sensitivity analysis, to provide an overall estimate, the four quality assessment scores of each MA will then be converted into a score from 0-100 by dividing the individual score by the maximum possible score for that assessment and multiplying by 100. An overall quality score will be calculated for each MA as the mean of the four individual quality assessment scores**.** If a MA scores “not applicable” in all categories of the PRESS score (ie. did not provide a search strategy), the PRESS score will not included in the calculation of the overall quality score, which in this situation will be determined as the average of the other 3 quality assessments.

***Statistical analysis***

The Wilks-Shapiro test will be used to assess normality for the final checklist scores. For each checklist, the mean and standard deviation (SD) or the median and the inter-quartile ranges (IQR) shall be calculated for normally and non-normally distributed scores respectively. Descriptive bivariate analysis will be used to compare each score against binary variables using the Student’s T-test for normally distributed scores and Mann Whitney U test for non-normally distributed scores. For categorical variables with more than 2 levels, ANOVA and Kruskal Wallis Test will be used for normally and non-normally distributed scores, respectively. Correlation between continuous variables and scores will also calculated and reported as Pearson’s correlation coefficient (r).

For PRESS checklist, Cohen’s Kappa value for inter-observer agreement between librarians will be determined due to its subjective evaluation. The Kappa result shall be interpreted as follows: values ≤ 0 as indicating no agreement and 0.01–0.20 as none to slight, 0.21–0.40 as fair, 0.41– 0.60 as moderate, 0.61–0.80 as substantial, and 0.81–1.00 as almost perfect agreement.

Multiple linear regression will be conducted on the following variables to identify factors associated with each of the standard checklists (PRESS, PRISMA, and IOM) as well as the overall score: librarian Involvement, clinical field, compliance with journal guidelines, impact factor (Clarivate Analytics), MA of RCT only, network MA, and number of included studies.

The clinical field associated with highest mean scores will be the reference in the regression models.

P value < 0.05 will be considered statistically significant and 95% confidence intervals will be calculated. Analyses were performed using R (version 3.3.3 R Project for Statistical Computing) within RStudio (0.99.489, http://www.rstudio.com).
