# Supplementary material for: An assessment of the quality of current clinical meta-analyses
Source: BMC Med Res Methodol. 2020 May 7;20:105. doi: 10.1186/s12874-020-00999-9 (PMC7204021; doi:10.1186/s12874-020-00999-9)
Supplement: Supplementary file 1 — Additional file 1: Table S1. Summary of quality assessments and scoring Study Protocol. Table S2. Top tier clinical journals (SCImago ranking) reporting meta-analyses. Table S3. The highest-ranking clinical journals (SCImago) reporting aggregate-level meta-analyses and the number of included meta-analyses in our quality assessment. Table S4. Peer Review of Electronic Search Strategies (PRESS) checklist scoring by two librarians. For the points in each section of the PRESS checklist, if none or less than half were addressed, it was scored as “not addressed” or “mostly incomplete.” If half, more than half, or all were addressed, it was scored as “mostly complete” or “fully addressed.” “Not applicable” was used if the point was not relevant to the instance or unable to be evaluated. Table S5. Preferred Reporting Items for Systematic Reviews and Meta-Analyses (PRISMA) Checklist scoring. Table S6. Institute of Medicine (IOM) checklist scoring. For the points in each section of the IOM checklist, if none or less than half were addressed, it was scored as “not addressed” or “mostly incomplete.” If half, more than half, or all were addressed, it was scored as “mostly complete” or “fully addressed.” “Not applicable” was used if the point was not relevant to the instance or unable to be evaluated. Table S7. Inter-observer agreement between librarians for Peer Review of Electronic Search Strategies (PRESS) checklist. Table S8. Correlation (r) between continuous meta-analyses variables and checklist scores. [file 12874_2020_999_MOESM1_ESM.docx]

**Supplementary Table 1.** Summary of quality assessments and scoring

| **Quality Assessment** | **Maximum possible number of checklist/scoring items** | | **Scoring** | **Mean (SD)** | **Median (IQR)** |
| --- | --- | --- | --- | --- | --- |
| **PRESS** | 6 | 0 – not applicable  1 – not addressed  2 – mostly incomplete  3 – mostly complete  4 – fully addressed | | 61.6 (19.1) | 62.5 [45.8, 75.0] |
| **PRISMA** | 27 | 0 – not reported  1 – reported | | 91.6 (8.4) | 92.6 [88.9, 96.3] |
| **IOM** | 16 | 0 – not applicable  1 – not addressed  2 – mostly incomplete  3 – mostly complete  4 – fully addressed | | 81.0 (8.2) | 81.3 [76.6, 85.9] |
| **Cochrane Quality Items** | 6 | 0 – not reported  1 – reported | | 63.2 (22.2) | 66.7 [50.0, 83.3] |
| **Overall score** | 4 | mean percentage of the four individual scoring items described above | | 74.4 (8.5) | 75.0 [69.2, 80.7] |

IOM: Institute of Medicine; IQR- interquartile range; PRESS: Peer Review of Electronic Search Strategies; PRISMA: Preferred Reporting Items for Systematic Reviews and Meta-Analyses, SD: Standard deviation.

**Supplementary Table 2.** Top tier clinical journals (SCImago ranking) reporting meta-analyses.

| Journal | Published meta-analyses  (2016-2017) | Published patient-level meta-analyses  (2016-2017) | Published > 10 study-level clinical meta-analyses (2016-2017) |
| --- | --- | --- | --- |
| [CA - A Cancer Journal for Clinicians](https://www.scimagojr.com/journalsearch.php?q=28773&tip=sid&clean=0) | **+** | **-** | **-** |
| [New England Journal of Medicine](https://www.scimagojr.com/journalsearch.php?q=15847&tip=sid&clean=0) | **+** | **+** | **-** |
| Lancet | **+** | **+** | **+** |
| Cancer Cell | **+** | **+** | **-** |
| Journal of the American College of Cardiology | **+** | **+** | **+** |
| Journal of Clinical Oncology | **+** | **+** | **-** |
| Science Translational Medicine | **+** | **-** | **-** |
| European Heart Journal | **+** | **+** | **+** |
| [Circulation](https://www.scimagojr.com/journalsearch.php?q=22581&tip=sid&clean=0) | **+** | **+** | **+** |
| Journal of the American Medical Association | **+** | **+** | **+** |
| [Gastroenterology](https://www.scimagojr.com/journalsearch.php?q=28330&tip=sid&clean=0) | **+** | **+** | **+** |
| [Annals of the Rheumatic Diseases](https://www.scimagojr.com/journalsearch.php?q=19136&tip=sid&clean=0) | **+** | **+** | **+** |
| [Journal of Clinical Investigation](https://www.scimagojr.com/journalsearch.php?q=15870&tip=sid&clean=0) | **+** | **-** | **-** |
| [Acta Neuropathologica](https://www.scimagojr.com/journalsearch.php?q=17552&tip=sid&clean=0) | **+** | **-** | **-** |
| [Annals of Internal Medicine](https://www.scimagojr.com/journalsearch.php?q=26574&tip=sid&clean=0) | **+** | **+** | **+** |
| Gut | **+** | **+** | **+** |
| American Journal of Human Genetics | **+** | **-** | **-** |
| World Psychiatry | **+** | **-** | **+** |
| European Urology | **+** | **+** | **+** |
| Diabetes Care | **+** | **+** | **+** |
| Blood | **+** | **-** | **+** |
| American Journal of Respiratory and Critical Care Medicine | **+** | **+** | **-** |
| PLoS Medicine | **+** | **+** | **+** |
| European Journal of Heart Failure | **+** | **+** | **+** |
| Annals of Neurology | **+** | **+** | **+** |

**Supplementary Table 3**. The highest-ranking clinical journals (SCImago) reporting aggregate-level meta-analyses and the number of included meta-analyses in our quality assessment.

| **Journal** | **Number of included study level meta-analyses** | **SCImago Journal Scopus Database Ranking** | **Clarivate Analytics 2017 Impact Factor** | **Detailed author guidelines for meta-analyses available?** |
| --- | --- | --- | --- | --- |
| Lancet | 7 | 14.934 | 53.254 | Yes |
| Journal of American College of Cardiology | 11 | 11.061 | 16.834 | No |
| European Heart Journal | 13 | 9.315 | 23.425 | No |
| Circulation | 10 | 8.950 | 18.880 | Yes |
| The Journal of the American Medical Association | 10 | 8.876 | 47.661 | Yes |
| Gastroenterology | 12 | 7.958 | 20.773 | No |
| Annals of the Rheumatic Diseases | 10 | 7.699 | 12.350 | No |
| Annals of Internal Medicine | 12 | 7.466 | 19.384 | Yes |
| Gut | 8 | 7.440 | 17.016 | No |
| World Psychiatry | 7 | 7.027 | 30.000 | No |

**Supplementary Table 4.** Peer Review of Electronic Search Strategies (PRESS) checklist scoring by two librarians. For the points in each section of the PRESS checklist, if none or less than half were addressed, it was scored as “not addressed” or “mostly incomplete.” If half, more than half, or all were addressed, it was scored as “mostly complete” or “fully addressed.” “Not applicable” was used if the point was not relevant to the instance or unable to be evaluated.

| Checklist item | Number of meta-analyses fulfilling item (%) |
| --- | --- |
| TRANSLATION OF THE RESEARCH QUESTION Librarian 1 (%) |  |
| - not applicable | 4 (4) |
| - Not addressed | 5 (5) |
| - mostly incomplete | 29 (29) |
| - mostly complete | 33 (33) |
| - fully addressed | 29 (29) |
| TRANSLATION OF THE RESEARCH QUESTION Librarian 2 (%) |  |
| - not applicable | 3 (3) |
| - Not addressed | 5 (5) |
| - mostly incomplete | 31 (31) |
| - mostly complete | 35 (35) |
| - fully addressed | 26 (26) |
| BOOLEAN AND PROXIMITY OPERATORS Librarian 1 (%) |  |
| - not applicable | 8 (8) |
| - not addressed | 14 (14) |
| - mostly incomplete | 22 (22) |
| - mostly complete | 19 (19) |
| - fully addressed | 37 (37) |
| BOOLEAN AND PROXIMITY OPERATORS Librarian 2 (%) |  |
| - not applicable | 5 (5) |
| - not addressed | 17 (17) |
| - mostly incomplete | 21 (21) |
| - mostly complete | 28 (28) |
| - fully addressed | 29 (29) |
| SUBJECT HEADINGS Librarian 1 (%) |  |
| - not applicable | 4 (4) |
| - not addressed | 35 (35) |
| - mostly incomplete | 20 (20) |
| - mostly complete | 19 (19) |
| - fully addressed | 22 (22) |
| SUBJECT HEADINGS Librarian 2 (%) |  |
| - not applicable | 3 (3) |
| - not addressed | 30 (30) |
| - mostly incomplete | 24 (24) |
| - mostly complete | 20 (20) |
| - fully addressed | 23 (23) |
| TEXT WORD SEARCHING Librarian 1 (%) |  |
| - not applicable | 4 (4) |
| - not addressed | 5 (5) |
| - mostly incomplete | 48 (48) |
| - mostly complete | 27 (27) |
| - fully addressed | 16 (16) |
| TEXT WORD SEARCHING Librarian 2 (%) |  |
| - not applicable | 3 (3) |
| - not addressed | 7 (7) |
| - mostly incomplete | 52 (52) |
| - mostly complete | 25 (25) |
| - fully addressed | 13 (13) |
| SPELLING SYNTAX AND LINE NUMBERS Librarian 1 (%) |  |
| - not applicable | 12 (12) |
| - not addressed | 15 (15) |
| - mostly incomplete | 24 (24) |
| - mostly complete | 19 (19) |
| - fully addressed | 30 (30) |
| SPELLING SYNTAX AND LINE NUMBERS Librarian 2 (%) |  |
| - not applicable | 9 (9) |
| - not addressed | 24 (24) |
| - mostly incomplete | 17 (17) |
| - mostly complete | 16 (16) |
| - fully addressed | 34 (34) |
| LIMITS AND FILTERS Librarian 1 (%) |  |
| - not applicable | 28 (28) |
| - not addressed | 8 (8) |
| - mostly incomplete | 20 (20) |
| - mostly complete | 32 (32) |
| - fully addressed | 12 (12) |
| LIMITS AND FILTERS Librarian 2 (%) |  |
| - not applicable | 28 (28) |
| - not addressed | 11 (11) |
| - mostly incomplete | 26 (26) |
| - mostly complete | 17 (17) |
| - fully addressed | 18 (18) |

**Supplementary Table 5**. Preferred Reporting Items for Systematic Reviews and Meta-Analyses (PRISMA) Checklist scoring.

| Checklist Item | Number of meta-analyses reporting item (%) |
| --- | --- |
| Title | 92 (92) |
| Structured Summary | 99 (99) |
| Rationale | 100 (100) |
| Objectives | 99 (99) |
| Protocol Registration | 34 (34) |
| Eligibility Criteria | 98 (98) |
| Information Sources | 95 (95) |
| Search | 75 (75) |
| Study Selection | 86 (86) |
| Data Collection Process | 91 (91) |
| Data Items | 97 (97) |
| Risk of Bias in Individual Studies | 85 (85) |
| Summary of Measures | 100 (100) |
| Synthesis of Results | 98 (98) |
| Risk of Bias Across Studies | 86 (86) |
| Additional Analyses | 92 (92) |
| Study Selection | 98 (98) |
| Study Characteristics | 90 (90) |
| Risk of Bias Within Studies | 86 (86) |
| Results of Individual Studies | 91 (91) |
| Synthesis of Results | 99 (99) |
| Risk of Bias Across Studies | 86 (86) |
| Additional Analysis | 96 (96) |
| Summary of Evidence | 99 (99) |
| Limitations | 100 (100) |
| Conclusions | 100 (100) |
| Funding | 100 (100) |
|  |  |

**Supplementary Table 6**. Institute of Medicine (IOM) checklist scoring. For the points in each section of the IOM checklist, if none or less than half were addressed, it was scored as “not addressed” or “mostly incomplete.” If half, more than half, or all were addressed, it was scored as “mostly complete” or “fully addressed.” “Not applicable” was used if the point was not relevant to the instance or unable to be evaluated.

| Checklist item | Number of meta-analyses fulfilling item (%) |
| --- | --- |
| Standard 2.1 - Establish a team with appropriate expertise and experience to conduct the systematic review |  |
| - not applicable | 0 (0) |
| - not addressed | 0 (0) |
| - mostly incomplete | 0 (0) |
| - mostly complete | 0 (0) |
| - fully addressed | 100 (100) |
| Standard 2.2 - Manage bias and conflict of interest (COI) of the team conducting the systematic review |  |
| - not applicable | 0 (0) |
| - not addressed | 0 (0) |
| - mostly incomplete | 0 (0) |
| - mostly complete | 0 (0) |
| - fully addressed | 100 (100) |
| Standard 2.4 - Manage bias and COI for individuals providing input into the systematic review |  |
| - not applicable | 100 (0) |
| - not addressed | 0 (0) |
| - mostly incomplete | 0 (0) |
| - mostly complete | 0 (0) |
| - fully addressed | 0 (0) |
| Standard 2.5 - Formulate the topic for the systematic review |  |
| - not applicable | 0 (0) |
| - not addressed | 0 (0) |
| - mostly incomplete | 0 (0) |
| - mostly complete | 0 (0) |
| - fully addressed | 100 (100) |
| Standard 2.8 - Make the final protocol publicly available, and add any amendments to the protocol in a timely fashion |  |
| - not applicable | 0 (0) |
| - not addressed | 69 (69) |
| - mostly incomplete | 4 (4) |
| - mostly complete | 3 (3) |
| - fully addressed | 24 (24) |
| Standard 3.1 - Conduct a comprehensive systematic search for evidence |  |
| - not applicable | 0 (0) |
| - not addressed | 6 (6) |
| - mostly incomplete | 51 (51) |
| - mostly complete | 29 (29) |
| - fully addressed | 14 (14) |
| Standard 3.2 - Take action to address potentially biased reporting of research results |  |
| - not applicable | 0 (0) |
| - not addressed | 13 (13) |
| - mostly incomplete | 27 (27) |
| - mostly complete | 38 (38) |
| - fully addressed | 22 (22) |
| Standard 3.3 - Screen and select studies |  |
| - not applicable | 0 (0) |
| - not addressed | 4 (4) |
| - mostly incomplete | 14 (14) |
| - mostly complete | 36 (36) |
| - fully addressed | 46 (46) |
| Standard 3.4 - Document the search |  |
| - not applicable | 1 (1) |
| - not addressed | 22 (22) |
| - mostly incomplete | 23 (23) |
| - mostly complete | 26 (26) |
| - fully addressed | 28 (28) |
| Standard 3.5 - Manage data collection |  |
| - not applicable | 0 (0) |
| - not addressed | 3 (3) |
| - mostly incomplete | 9 ( 9) |
| - mostly complete | 33 (33) |
| - fully addressed | 55 (55) |
| Standard 3.6 - Critically appraise each study |  |
| - not applicable | 0 (0) |
| - not addressed | 6 (6) |
| - mostly incomplete | 3 (3) |
| - mostly complete | 26 (26) |
| - fully addressed | 65 (65) |
| Standard 4.1 - Use a prespecified method to evaluate the body of evidence |  |
| - not applicable | 0 (0) |
| - not addressed | 13 (13) |
| - mostly incomplete | 1 (1) |
| - mostly complete | 12 (12) |
| - fully addressed | 74 (74) |
| Standard 4.2 - Conduct a qualitative synthesis |  |
| - Not applicable | 0 (0) |
| - Not addressed | 4 (4) |
| - mostly incomplete | 1 (1) |
| - mostly complete | 38 (38) |
| - fully addressed | 57 (57) |
| Standard 4.3 - Decide if, in addition to a qualitative analysis, the systematic review will include a quantitative analysis (meta-analysis) |  |
| - not applicable | 0 (0) |
| - not addressed | 0 (0) |
| - mostly incomplete | 0 (0) |
| - mostly complete | 1(1) |
| - fully addressed | 99(99) |
| Standard 4.4 Conduct of meta-analysis |  |
| - not applicable | 0 (0) |
| - not addressed | 1 (1) |
| - mostly incomplete | 0 (0) |
| - mostly complete | 1 (1) |
| - fully addressed | 98 (98) |
| Standard 5.1 - Prepare final report using a structured format |  |
| - not applicable | 0 (0) |
| - not addressed | 1 (1) |
| - mostly incomplete | 14 (14) |
| - mostly complete | 53 (53) |
| - fully addressed | 32 (32) |

**Supplementary Table** 7. Inter-observer agreement between librarians for Peer Review of Electronic Search Strategies (PRESS) checklist.

| 1. **TRANSLATION OF THE RESEARCH QUESTION** | | | | | | | | | |
| --- | --- | --- | --- | --- | --- | --- | --- | --- | --- |
| **Librarian 1** | | | | | | | | | |
| **Librarian 2** |  | 0 | 1 | 2 | 3 | 4 | **Total** | **Kappa** | **P-value** |
|  | 0 | 1 | 1 | 0 | 1 | 0 | 3 | 0.55 | <0.001 |
|  |  | 25.0% | 20.0% | 0.0% | 3.0% | 0.0% | 3.0% |  |  |
|  | 1 | 0 | 3 | 1 | 0 | 1 | 5 |  |  |
|  |  | 0.0% | 60.0% | 3.4% | 0.0% | 3.4% | 5.0% |  |  |
|  | 2 | 1 | 1 | 21 | 4 | 4 | 31 |  |  |
|  |  | 25.0% | 20.0% | 72.4% | 12.1% | 13.8% | 31.0% |  |  |
|  | 3 | 2 | 0 | 6 | 23 | 4 | 35 |  |  |
|  |  | 50.0% | 0.0% | 20.7% | 69.7% | 13.8% | 35.0% |  |  |
|  | 4 | 0 | 0 | 1 | 5 | 20 | 26 |  |  |
|  |  | 0.0% | 0.0% | 3.4% | 15.2% | 69.0% | 26.0% |  |  |
| **Total** | | 4 | 5 | 29 | 33 | 29 | 100 |  |  |
|  |  | 100.0% | 100.0% | 100.0% | 100.0% | 100.0% | 100.0% |  |  |
| 2. **BOOLEAN AND PROXIMITY OPERATORS** | | | | | | | | | |
| **Librarian 1** | | | | | | | | | |
| **Librarian 2** |  | 0 | 1 | 2 | 3 | 4 | **Total** | **Kappa** | **P-value** |
|  | 0 | 3 | 1 | 0 | 0 | 1 | 5 | 0.49 | <0.001 |
|  |  | 37.5% | 7.1% | 0.0% | 0.0% | 2.7% | 5.0% |  |  |
|  | 1 | 4 | 10 | 3 | 0 | 0 | 17 |  |  |
|  |  | 50.0% | 71.4% | 13.6% | 0.0% | 0.0% | 17.0% |  |  |
|  | 2 | 0 | 1 | 13 | 3 | 4 | 21 |  |  |
|  |  | 0.0% | 7.1% | 59.1% | 15.8% | 10.8% | 21.0% |  |  |
|  | 3 | 0 | 1 | 4 | 13 | 10 | 28 |  |  |
|  |  | 0.0% | 7.1% | 18.2% | 68.4% | 27.0% | 28.0% |  |  |
|  | 4 | 1 | 1 | 2 | 3 | 22 | 29 |  |  |
|  |  | 12.5% | 7.1% | 9.1% | 15.8% | 59.5% | 29.0% |  |  |
| **Total** | | 8 | 14 | 22 | 19 | 37 | 100 |  |  |
|  |  | 100.0% | 100.0% | 100.0% | 100.0% | 100.0% | 100.0% |  |  |
| 3. **SUBJECT HEADINGS** | | | | | | | | | |
| **Librarian 1** | | | | | | | | | |
| **Librarian 2** |  | 0 | 1 | 2 | 3 | 4 | **Total** | **Kappa** | **P-value** |
|  | 0 | 1 | 1 | 1 | 0 | 0 | 3 | 0.55 | <0.001 |
|  |  | 25.0% | 2.9% | 5.0% | 0.0% | 0.0% | 3.0% |  |  |
|  | 1 | 2 | 25 | 3 | 0 | 0 | 30 |  |  |
|  |  | 50.0% | 71.4% | 15.0% | 0.0% | 0.0% | 30.0% |  |  |
|  | 2 | 0 | 8 | 12 | 4 | 0 | 24 |  |  |
|  |  | 0.0% | 22.9% | 60.0% | 21.1% | 0.0% | 24.0% |  |  |
|  | 3 | 1 | 0 | 3 | 11 | 5 | 20 |  |  |
|  |  | 25.0% | 0.0% | 15.0% | 57.9% | 22.7% | 20.0% |  |  |
|  | 4 | 0 | 1 | 1 | 4 | 17 | 23 |  |  |
|  |  | 0.0% | 2.9% | 5.0% | 21.1% | 77.3% | 23.0% |  |  |
| **Total** | | 4 | 35 | 20 | 19 | 22 | 100 |  |  |
|  |  | 100.0% | 100.0% | 100.0% | 100.0% | 100.0% | 100.0% |  |  |
| 4. **TEXT WORD SEARCHING** | | | | | | | | | |
| **Librarian 1** | | | | | | | | | |
| **Librarian 2** |  | 0 | 1 | 2 | 3 | 4 | **Total** | **Kappa** | **P-value** |
|  | 0 | 1 | 1 | 1 | 0 | 0 | 3 | 0.30 | <0.001 |
|  |  | 25.0% | 20.0% | 2.1% | 0.0% | 0.0% | 3.0% |  |  |
|  | 1 | 0 | 3 | 4 | 0 | 0 | 7 |  |  |
|  |  | 0.0% | 60.0% | 8.3% | 0.0% | 0.0% | 7.0% |  |  |
|  | 2 | 3 | 1 | 31 | 13 | 4 | 52 |  |  |
|  |  | 75.0% | 20.0% | 64.6% | 48.1% | 25.0% | 52.0% |  |  |
|  | 3 | 0 | 0 | 8 | 12 | 5 | 25 |  |  |
|  |  | 0.0% | 0.0% | 16.7% | 44.4% | 31.3% | 25.0% |  |  |
|  | 4 | 0 | 0 | 4 | 2 | 7 | 13 |  |  |
|  |  | 0.0% | 0.0% | 8.3% | 7.4% | 43.8% | 13.0% |  |  |
| **Total** | | 4 | 5 | 48 | 27 | 16 | 100 |  |  |
|  |  | 100.0% | 100.0% | 100.0% | 100.0% | 100.0% | 100.0% |  |  |
| 5. **SPELLING, SYNTAX, AND LINE NUMBERS** | | | | | | | | | |
| **Librarian 1** | | | | | | | | | |
| **Librarian 2** |  | 0 | 1 | 2 | 3 | 4 | **Total** | **Kappa** | **P-value** |
|  | 0 | 5 | 1 | 2 | 1 | 0 | 9 | 0.54 | <0.001 |
|  |  | 41.7% | 6.7% | 8.3% | 5.3% | 0.0% | 9.0% |  |  |
|  | 1 | 3 | 13 | 6 | 1 | 1 | 24 |  |  |
|  |  | 25.0% | 86.7% | 25.0% | 5.3% | 3.3% | 24.0% |  |  |
|  | 2 | 3 | 1 | 10 | 1 | 2 | 17 |  |  |
|  |  | 25.0% | 6.7% | 41.7% | 5.3% | 6.7% | 17.0% |  |  |
|  | 3 | 0 | 0 | 1 | 12 | 3 | 16 |  |  |
|  |  | 0.0% | 0.0% | 4.2% | 63.2% | 10.0% | 16.0% |  |  |
|  | 4 | 1 | 0 | 5 | 4 | 24 | 34 |  |  |
|  |  | 8.3% | 0.0% | 20.8% | 21.1% | 80.0% | 34.0% |  |  |
| **Total** | | 12 | 15 | 24 | 19 | 30 | 100 |  |  |
|  |  | 100.0% | 100.0% | 100.0% | 100.0% | 100.0% | 100.0% |  |  |
| 6. **LIMITS AND FILTERS** | | | | | | | | | |
| **Librarian 1** | | | | | | | | | |
| **Librarian 2** |  | 0 | 1 | 2 | 3 | 4 | **Total** | **Kappa** | **P-value** |
|  | 0 | 20 | 3 | 0 | 2 | 3 | 28 | 0.43 | <0.001 |
|  |  | 71.4% | 37.5% | 0.0% | 6.3% | 25.0% | 28.0% |  |  |
|  | 1 | 3 | 5 | 1 | 2 | 0 | 11 |  |  |
|  |  | 10.7% | 62.5% | 5.0% | 6.3% | 0.0% | 11.0% |  |  |
|  | 2 | 3 | 0 | 13 | 8 | 2 | 26 |  |  |
|  |  | 10.7% | 0.0% | 65.0% | 25.0% | 16.7% | 26.0% |  |  |
|  | 3 | 1 | 0 | 4 | 11 | 1 | 17 |  |  |
|  |  | 3.6% | 0.0% | 20.0% | 34.4% | 8.3% | 17.0% |  |  |
|  | 4 | 1 | 0 | 2 | 9 | 6 | 18 |  |  |
|  |  | 3.6% | 0.0% | 10.0% | 28.1% | 50.0% | 18.0% |  |  |
| **Total** | | 28 | 8 | 20 | 32 | 12 | 100 |  |  |
|  |  | 100.0% | 100.0% | 100.0% | 100.0% | 100.0% | 100.0% |  |  |

**Supplementary Table 8.** Correlation (r) between continuous meta-analyses variables and checklist scores.

| Variable | PRESS | PRISMA | IOM | Overall |
| --- | --- | --- | --- | --- |
| Number of included studies | 0.09 | -0.01 | 0.03 | -0.04 |
| P-value | 0.41 | 0.95 | 0.77 | 0.72 |
| Journal Impact Factor (2017 Clarivate) | -0.11 | 0.09 | 0.12 | -0.03 |
| P-value | 0.30 | 0.38 | 0.23 | 0.78 |
| Journal ScImago Ranking | -0.08 | -0.02 | 0.01 | -0.06 |
| P-value | 0.45 | 0.85 | 0.94 | 0.58 |

IOM: Institute of Medicine; PRESS: Peer Review of Electronic Search Strategies; PRISMA: Preferred Reporting Items for Systematic Reviews and Meta-Analyses.

**References of included meta-analyses**

1.	Aldridge RW, Story A, Hwang SW, Nordentoft M, Luchenski SA, Hartwell G, et al. Morbidity and mortality in homeless individuals, prisoners, sex workers, and individuals with substance use disorders in high-income countries: a systematic review and meta-analysis. Lancet (London, England). 2018;391(10117):241-50.

2.	Alibhai SMH, Zukotynski K, Walker-Dilks C, Emmenegger U, Finelli A, Morgan SC, et al. Bone Health and Bone-Targeted Therapies for Nonmetastatic Prostate Cancer: A Systematic Review and Meta-analysis. Ann Intern Med. 2017;167(5):341-50.

3.	Anderson L, Oldridge N, Thompson DR, Zwisler A-D, Rees K, Martin N, et al. Exercise-Based Cardiac Rehabilitation for Coronary Heart Disease: Cochrane Systematic Review and Meta-Analysis. J Am Coll Cardiol. 2016;67(1):1-12.

4.	Angermeyer MC, van der Auwera S, Carta MG, Schomerus G. Public attitudes towards psychiatry and psychiatric treatment at the beginning of the 21st century: a systematic review and meta-analysis of population surveys. World psychiatry : official journal of the World Psychiatric Association (WPA). 2017;16(1):50-61.

5.	Auffret V, Regueiro A, Del Trigo M, Abdul-Jawad Altisent O, Campelo-Parada F, Chiche O, et al. Predictors of Early Cerebrovascular Events in Patients With Aortic Stenosis Undergoing Transcatheter Aortic Valve Replacement. J Am Coll Cardiol. 2016;68(7):673-84.

6.	Aune D, Sen A, Norat T, Janszky I, Romundstad P, Tonstad S, et al. Body Mass Index, Abdominal Fatness, and Heart Failure Incidence and Mortality: A Systematic Review and Dose-Response Meta-Analysis of Prospective Studies. Circulation. 2016;133(7):639-49.

7.	Baekgaard JS, Viereck S, Moller TP, Ersboll AK, Lippert F, Folke F. The Effects of Public Access Defibrillation on Survival After Out-of-Hospital Cardiac Arrest: A Systematic Review of Observational Studies. Circulation. 2017;136(10):954-65.

8.	Bailie L, Loughrey MB, Coleman HG. Lifestyle Risk Factors for Serrated Colorectal Polyps: A Systematic Review and Meta-analysis. Gastroenterology. 2017;152(1):92-104.

9.	Baumgartner C, da Costa BR, Collet T-H, Feller M, Floriani C, Bauer DC, et al. Thyroid Function Within the Normal Range, Subclinical Hypothyroidism, and the Risk of Atrial Fibrillation. Circulation. 2017;136(22):2100-16.

10.	Bennaghmouch N, de Veer AJWM, Bode K, Mahmoodi BK, Dewilde WJM, Lip GYH, et al. Efficacy and Safety of the Use of Non-Vitamin K Antagonist Oral Anticoagulants in Patients With Nonvalvular Atrial Fibrillation and Concomitant Aspirin Therapy: A Meta-Analysis of Randomized Trials. Circulation. 2018;137(11):1117-29.

11.	Bikdeli B, Chatterjee S, Desai NR, Kirtane AJ, Desai MM, Bracken MB, et al. Inferior Vena Cava Filters to Prevent Pulmonary Embolism: Systematic Review and Meta-Analysis. J Am Coll Cardiol. 2017;70(13):1587-97.

12.	Bittl JA, Baber U, Bradley SM, Wijeysundera DN. Duration of Dual Antiplatelet Therapy: A Systematic Review for the 2016 ACC/AHA Guideline Focused Update on Duration of Dual Antiplatelet Therapy in Patients With Coronary Artery Disease: A Report of the American College of Cardiology/American Heart Association Task Force on Clinical Practice Guidelines. J Am Coll Cardiol. 2016;68(10):1116-39.

13.	Cassese S, Byrne RA, Ndrepepa G, Kufner S, Wiebe J, Repp J, et al. Everolimus-eluting bioresorbable vascular scaffolds versus everolimus-eluting metallic stents: a meta-analysis of randomised controlled trials. Lancet (London, England). 2016;387(10018):537-44.

14.	Chou R, Dana T, Blazina I, Daeges M, Jeanne TL. Statins for Prevention of Cardiovascular Disease in Adults: Evidence Report and Systematic Review for the US Preventive Services Task Force. JAMA. 2016;316(19):2008-24.

15.	Chou R, Dana T, Bougatsos C, Grusing S, Blazina I. Screening for Impaired Visual Acuity in Older Adults: Updated Evidence Report and Systematic Review for the US Preventive Services Task Force. JAMA. 2016;315(9):915-33.

16.	Cipriani A, Zhou X, Del Giovane C, Hetrick SE, Qin B, Whittington C, et al. Comparative efficacy and tolerability of antidepressants for major depressive disorder in children and adolescents: a network meta-analysis. Lancet (London, England). 2016;388(10047):881-90.

17.	Collet C, Asano T, Miyazaki Y, Tenekecioglu E, Katagiri Y, Sotomi Y, et al. Late thrombotic events after bioresorbable scaffold implantation: a systematic review and meta-analysis of randomized clinical trials. Eur Heart J. 2017;38(33):2559-66.

18.	Collister D, Komenda P, Hiebert B, Gunasekara R, Xu Y, Eng F, et al. The Effect of Erythropoietin-Stimulating Agents on Health-Related Quality of Life in Anemia of Chronic Kidney Disease: A Systematic Review and Meta-analysis. Ann Intern Med. 2016;164(7):472-8.

19.	Correll CU, Solmi M, Veronese N, Bortolato B, Rosson S, Santonastaso P, et al. Prevalence, incidence and mortality from cardiovascular disease in patients with pooled and specific severe mental illness: a large-scale meta-analysis of 3,211,768 patients and 113,383,368 controls. World psychiatry : official journal of the World Psychiatric Association (WPA). 2017;16(2):163-80.

20.	Cuijpers P, Cristea IA, Karyotaki E, Reijnders M, Huibers MJH. How effective are cognitive behavior therapies for major depression and anxiety disorders? A meta-analytic update of the evidence. World psychiatry : official journal of the World Psychiatric Association (WPA). 2016;15(3):245-58.

21.	D'Ascenzo F, Iannaccone M, Saint-Hilary G, Bertaina M, Schulz-Schupke S, Wahn Lee C, et al. Impact of design of coronary stents and length of dual antiplatelet therapies on ischaemic and bleeding events: a network meta-analysis of 64 randomized controlled trials and 102 735 patients. Eur Heart J. 2017;38(42):3160-72.

22.	D'Souza R, Ostro J, Shah PS, Silversides CK, Malinowski A, Murphy KE, et al. Anticoagulation for pregnant women with mechanical heart valves: a systematic review and meta-analysis. Eur Heart J. 2017;38(19):1509-16.

23.	da Costa BR, Reichenbach S, Keller N, Nartey L, Wandel S, Juni P, et al. Effectiveness of non-steroidal anti-inflammatory drugs for the treatment of pain in knee and hip osteoarthritis: a network meta-analysis. Lancet (London, England). 2017;390(10090):e21-e33.

24.	Dawes AJ, Maggard-Gibbons M, Maher AR, Booth MJ, Miake-Lye I, Beroes JM, et al. Mental Health Conditions Among Patients Seeking and Undergoing Bariatric Surgery: A Meta-analysis. JAMA. 2016;315(2):150-63.

25.	Dayan N, Filion KB, Okano M, Kilmartin C, Reinblatt S, Landry T, et al. Cardiovascular Risk Following Fertility Therapy: Systematic Review and Meta-Analysis. J Am Coll Cardiol. 2017;70(10):1203-13.

26.	Ebell MH, Call M, Shinholser J, Gardner J. Does This Patient Have Infectious Mononucleosis?: The Rational Clinical Examination Systematic Review. JAMA. 2016;315(14):1502-9.

27.	Eusebi LH, Ratnakumaran R, Yuan Y, Solaymani-Dodaran M, Bazzoli F, Ford AC. Global prevalence of, and risk factors for, gastro-oesophageal reflux symptoms: a meta-analysis. Gut. 2018;67(3):430-40.

28.	Evidence Review Committee M, Bittl JA, Baber U, Bradley SM, Wijeysundera DN. Duration of Dual Antiplatelet Therapy: A Systematic Review for the 2016 ACC/AHA Guideline Focused Update on Duration of Dual Antiplatelet Therapy in Patients With Coronary Artery Disease: A Report of the American College of Cardiology/American Heart Association Task Force on Clinical Practice Guidelines. Circulation. 2016;134(10):e156-78.

29.	Firth J, Torous J, Nicholas J, Carney R, Pratap A, Rosenbaum S, et al. The efficacy of smartphone-based mental health interventions for depressive symptoms: a meta-analysis of randomized controlled trials. World psychiatry : official journal of the World Psychiatric Association (WPA). 2017;16(3):287-98.

30.	Fisher OM, Lord SJ, Falkenback D, Clemons NJ, Eslick GD, Lord RV. The prognostic value of TP53 mutations in oesophageal adenocarcinoma: a systematic review and meta-analysis. Gut. 2017;66(3):399-410.

31.	Freiman JM, Tran TM, Schumacher SG, White LF, Ongarello S, Cohn J, et al. Hepatitis C Core Antigen Testing for Diagnosis of Hepatitis C Virus Infection: A Systematic Review and Meta-analysis. Ann Intern Med. 2016;165(5):345-55.

32.	Galling B, Roldan A, Hagi K, Rietschel L, Walyzada F, Zheng W, et al. Antipsychotic augmentation vs. monotherapy in schizophrenia: systematic review, meta-analysis and meta-regression analysis. World psychiatry : official journal of the World Psychiatric Association (WPA). 2017;16(1):77-89.

33.	Ganesan AN, Chew DP, Hartshorne T, Selvanayagam JB, Aylward PE, Sanders P, et al. The impact of atrial fibrillation type on the risk of thromboembolism, mortality, and bleeding: a systematic review and meta-analysis. Eur Heart J. 2016;37(20):1591-602.

34.	Gargiulo G, Sannino A, Capodanno D, Barbanti M, Buccheri S, Perrino C, et al. Transcatheter Aortic Valve Implantation Versus Surgical Aortic Valve Replacement: A Systematic Review and Meta-analysis. Ann Intern Med. 2016;165(5):334-44.

35.	Gili S, Grosso Marra W, D'Ascenzo F, Lonni E, Calcagno A, Cannillo M, et al. Comparative safety and efficacy of statins for primary prevention in human immunodeficiency virus-positive patients: a systematic review and meta-analysis. Eur Heart J. 2016;37(48):3600-9.

36.	Hall KD, Guo J. Obesity Energetics: Body Weight Regulation and the Effects of Diet Composition. Gastroenterology. 2017;152(7):1718-27.e3.

37.	Hassan C, Repici A, Sharma P, Correale L, Zullo A, Bretthauer M, et al. Efficacy and safety of endoscopic resection of large colorectal polyps: a systematic review and meta-analysis. Gut. 2016;65(5):806-20.

38.	Henaux S, Ruyssen-Witrand A, Cantagrel A, Barnetche T, Fautrel B, Filippi N, et al. Risk of losing remission, low disease activity or radiographic progression in case of bDMARD discontinuation or tapering in rheumatoid arthritis: systematic analysis of the literature and meta-analysis. Ann Rheum Dis. 2018;77(4):515-22.

39.	Hooi JKY, Lai WY, Ng WK, Suen MMY, Underwood FE, Tanyingoh D, et al. Global Prevalence of Helicobacter pylori Infection: Systematic Review and Meta-Analysis. Gastroenterology. 2017;153(2):420-9.

40.	Jinatongthai P, Kongwatcharapong J, Foo CY, Phrommintikul A, Nathisuwan S, Thakkinstian A, et al. Comparative efficacy and safety of reperfusion therapy with fibrinolytic agents in patients with ST-segment elevation myocardial infarction: a systematic review and network meta-analysis. Lancet (London, England). 2017;390(10096):747-59.

41.	Khan AR, Golwala H, Tripathi A, Bin Abdulhak AA, Bavishi C, Riaz H, et al. Impact of total occlusion of culprit artery in acute non-ST elevation myocardial infarction: a systematic review and meta-analysis. Eur Heart J. 2017;38(41):3082-9.

42.	Khera R, Murad MH, Chandar AK, Dulai PS, Wang Z, Prokop LJ, et al. Association of Pharmacological Treatments for Obesity With Weight Loss and Adverse Events: A Systematic Review and Meta-analysis. JAMA. 2016;315(22):2424-34.

43.	Klem F, Wadhwa A, Prokop LJ, Sundt WJ, Farrugia G, Camilleri M, et al. Prevalence, Risk Factors, and Outcomes of Irritable Bowel Syndrome After Infectious Enteritis: A Systematic Review and Meta-analysis. Gastroenterology. 2017;152(5):1042-54.e1.

44.	Korteland NM, Etnel JRG, Arabkhani B, Mokhles MM, Mohamad A, Roos-Hesselink JW, et al. Mechanical aortic valve replacement in non-elderly adults: meta-analysis and microsimulation. Eur Heart J. 2017;38(45):3370-7.

45.	Kunutsor SK, Seidu S, Khunti K. Statins and secondary prevention of venous thromboembolism: pooled analysis of published observational cohort studies. Eur Heart J. 2017;38(20):1608-12.

46.	Lee Y-C, Chiang T-H, Chou C-K, Tu Y-K, Liao W-C, Wu M-S, et al. Association Between Helicobacter pylori Eradication and Gastric Cancer Incidence: A Systematic Review and Meta-analysis. Gastroenterology. 2016;150(5):1113-24.e5.

47.	Lipinski MJ, Benedetto U, Escarcega RO, Biondi-Zoccai G, Lhermusier T, Baker NC, et al. The impact of proprotein convertase subtilisin-kexin type 9 serine protease inhibitors on lipid levels and outcomes in patients with primary hypercholesterolaemia: a network meta-analysis. Eur Heart J. 2016;37(6):536-45.

48.	Loffredo L, Pastori D, Farcomeni A, Violi F. Effects of Anticoagulants in Patients With Cirrhosis and Portal Vein Thrombosis: A Systematic Review and Meta-analysis. Gastroenterology. 2017;153(2):480-7.e1.

49.	Mariscalco G, Wozniak MJ, Dawson AG, Serraino GF, Porter R, Nath M, et al. Body Mass Index and Mortality Among Adults Undergoing Cardiac Surgery: A Nationwide Study With a Systematic Review and Meta-Analysis. Circulation. 2017;135(9):850-63.

50.	Maruthur NM, Tseng E, Hutfless S, Wilson LM, Suarez-Cuervo C, Berger Z, et al. Diabetes Medications as Monotherapy or Metformin-Based Combination Therapy for Type 2 Diabetes: A Systematic Review and Meta-analysis. Ann Intern Med. 2016;164(11):740-51.

51.	Mills KT, Obst KM, Shen W, Molina S, Zhang H-J, He H, et al. Comparative Effectiveness of Implementation Strategies for Blood Pressure Control in Hypertensive Patients: A Systematic Review and Meta-analysis. Ann Intern Med. 2018;168(2):110-20.

52.	Montone RA, Niccoli G, De Marco F, Minelli S, D'Ascenzo F, Testa L, et al. Temporal Trends in Adverse Events After Everolimus-Eluting Bioresorbable Vascular Scaffold Versus Everolimus-Eluting Metallic Stent Implantation: A Meta-Analysis of Randomized Controlled Trials. Circulation. 2017;135(22):2145-54.

53.	Mostofsky E, Chahal HS, Mukamal KJ, Rimm EB, Mittleman MA. Alcohol and Immediate Risk of Cardiovascular Events: A Systematic Review and Dose-Response Meta-Analysis. Circulation. 2016;133(10):979-87.

54.	Nelson AD, Camilleri M, Chirapongsathorn S, Vijayvargiya P, Valentin N, Shin A, et al. Comparison of efficacy of pharmacological treatments for chronic idiopathic constipation: a systematic review and network meta-analysis. Gut. 2017;66(9):1611-22.

55.	Nelson HD, Fu R, Cantor A, Pappas M, Daeges M, Humphrey L. Effectiveness of Breast Cancer Screening: Systematic Review and Meta-analysis to Update the 2009 U.S. Preventive Services Task Force Recommendation. Ann Intern Med. 2016;164(4):244-55.

56.	Pan Y, Chen W, Xu Y, Yi X, Han Y, Yang Q, et al. Genetic Polymorphisms and Clopidogrel Efficacy for Acute Ischemic Stroke or Transient Ischemic Attack: A Systematic Review and Meta-Analysis. Circulation. 2017;135(1):21-33.

57.	Parian A, Limketkai B, Koh J, Brant SR, Bitton A, Cho JH, et al. Appendectomy does not decrease the risk of future colectomy in UC: results from a large cohort and meta-analysis. Gut. 2017;66(8):1390-7.

58.	Paul S, Saxena A, Terrin N, Viveiros K, Balk EM, Wong JB. Hepatitis B Virus Reactivation and Prophylaxis During Solid Tumor Chemotherapy: A Systematic Review and Meta-analysis. Ann Intern Med. 2016;164(1):30-40.

59.	Petta S, Maida M, Macaluso FS, Barbara M, Licata A, Craxi A, et al. Hepatitis C Virus Infection Is Associated With Increased Cardiovascular Mortality: A Meta-Analysis of Observational Studies. Gastroenterology. 2016;150(1):145-6.

60.	Pinto-Sanchez MI, Causada-Calo N, Bercik P, Ford AC, Murray JA, Armstrong D, et al. Safety of Adding Oats to a Gluten-Free Diet for Patients With Celiac Disease: Systematic Review and Meta-analysis of Clinical and Observational Studies. Gastroenterology. 2017;153(2):395-409.e3.

61.	Popova S, Lange S, Shield K, Mihic A, Chudley AE, Mukherjee RAS, et al. Comorbidity of fetal alcohol spectrum disorder: a systematic review and meta-analysis. Lancet (London, England). 2016;387(10022):978-87.

62.	Rahmattulla C, Mooyaart AL, van Hooven D, Schoones JW, Bruijn JA, Dekkers OM, et al. Genetic variants in ANCA-associated vasculitis: a meta-analysis. Ann Rheum Dis. 2016;75(9):1687-92.

63.	Ramiro S, Sepriano A, Chatzidionysiou K, Nam JL, Smolen JS, van der Heijde D, et al. Safety of synthetic and biological DMARDs: a systematic literature review informing the 2016 update of the EULAR recommendations for management of rheumatoid arthritis. Ann Rheum Dis. 2017;76(6):1101-36.

64.	Reboussin DM, Allen NB, Griswold ME, Guallar E, Hong Y, Lackland DT, et al. Systematic Review for the 2017 ACC/AHA/AAPA/ABC/ACPM/AGS/APhA/ASH/ASPC/NMA/PCNA Guideline for the Prevention, Detection, Evaluation, and Management of High Blood Pressure in Adults: A Report of the American College of Cardiology/American Heart Association Task Force on Clinical Practice Guidelines. J Am Coll Cardiol. 2018;71(19):2176-98.

65.	Rempenault C, Combe B, Barnetche T, Gaujoux-Viala C, Lukas C, Morel J, et al. Metabolic and cardiovascular benefits of hydroxychloroquine in patients with rheumatoid arthritis: a systematic review and meta-analysis. Ann Rheum Dis. 2018;77(1):98-103.

66.	Renda G, Ricci F, Giugliano RP, De Caterina R. Non-Vitamin K Antagonist Oral Anticoagulants in Patients With Atrial Fibrillation and Valvular Heart Disease. J Am Coll Cardiol. 2017;69(11):1363-71.

67.	Sardar P, Chatterjee S, Aronow HD, Kundu A, Ramchand P, Mukherjee D, et al. Carotid Artery Stenting Versus Endarterectomy for Stroke Prevention: A Meta-Analysis of Clinical Trials. J Am Coll Cardiol. 2017;69(18):2266-75.

68.	Saver JL, Goyal M, van der Lugt A, Menon BK, Majoie CBLM, Dippel DW, et al. Time to Treatment With Endovascular Thrombectomy and Outcomes From Ischemic Stroke: A Meta-analysis. JAMA. 2016;316(12):1279-88.

69.	Schiattarella GG, Sannino A, Toscano E, Giugliano G, Gargiulo G, Franzone A, et al. Gut microbe-generated metabolite trimethylamine-N-oxide as cardiovascular risk biomarker: a systematic review and dose-response meta-analysis. Eur Heart J. 2017;38(39):2948-56.

70.	Schieir O, Tosevski C, Glazier RH, Hogg-Johnson S, Badley EM. Incident myocardial infarction associated with major types of arthritis in the general population: a systematic review and meta-analysis. Ann Rheum Dis. 2017;76(8):1396-404.

71.	Sepriano A, Rubio R, Ramiro S, Landewe R, van der Heijde D. Performance of the ASAS classification criteria for axial and peripheral spondyloarthritis: a systematic literature review and meta-analysis. Ann Rheum Dis. 2017;76(5):886-90.

72.	Shah MS, DeSantis TZ, Weinmaier T, McMurdie PJ, Cope JL, Altrichter A, et al. Leveraging sequence-based faecal microbial community survey data to identify a composite biomarker for colorectal cancer. Gut. 2018;67(5):882-91.

73.	Shankar-Hari M, Phillips GS, Levy ML, Seymour CW, Liu VX, Deutschman CS, et al. Developing a New Definition and Assessing New Clinical Criteria for Septic Shock: For the Third International Consensus Definitions for Sepsis and Septic Shock (Sepsis-3). JAMA. 2016;315(8):775-87.

74.	Shelton E, Laharie D, Scott FI, Mamtani R, Lewis JD, Colombel J-F, et al. Cancer Recurrence Following Immune-Suppressive Therapies in Patients With Immune-Mediated Diseases: A Systematic Review and Meta-analysis. Gastroenterology. 2016;151(1):97-109.e4.

75.	Shun-Shin MJ, Zheng SL, Cole GD, Howard JP, Whinnett ZI, Francis DP. Implantable cardioverter defibrillators for primary prevention of death in left ventricular dysfunction with and without ischaemic heart disease: a meta-analysis of 8567 patients in the 11 trials. Eur Heart J. 2017;38(22):1738-46.

76.	Silverman MG, Ference BA, Im K, Wiviott SD, Giugliano RP, Grundy SM, et al. Association Between Lowering LDL-C and Cardiovascular Risk Reduction Among Different Therapeutic Interventions: A Systematic Review and Meta-analysis. JAMA. 2016;316(12):1289-97.

77.	Silvester JA, Kurada S, Szwajcer A, Kelly CP, Leffler DA, Duerksen DR. Tests for Serum Transglutaminase and Endomysial Antibodies Do Not Detect Most Patients With Celiac Disease and Persistent Villous Atrophy on Gluten-free Diets: a Meta-analysis. Gastroenterology. 2017;153(3):689-701.e1.

78.	Siontis GCM, Praz F, Pilgrim T, Mavridis D, Verma S, Salanti G, et al. Transcatheter aortic valve implantation vs. surgical aortic valve replacement for treatment of severe aortic stenosis: a meta-analysis of randomized trials. Eur Heart J. 2016;37(47):3503-12.

79.	Smith TO, Zou K, Abdullah N, Chen X, Kingsbury SR, Doherty M, et al. Does flare trial design affect the effect size of non-steroidal anti-inflammatory drugs in symptomatic osteoarthritis? A systematic review and meta-analysis. Ann Rheum Dis. 2016;75(11):1971-8.

80.	Steinberg ZL, Dominguez-Islas CP, Otto CM, Stout KK, Krieger EV. Maternal and Fetal Outcomes of Anticoagulation in Pregnant Women With Mechanical Heart Valves. J Am Coll Cardiol. 2017;69(22):2681-91.

81.	Tan JA, Dehghan N, Chen W, Xie H, Esdaile JM, Avina-Zubieta JA. Mortality in ANCA-associated vasculitis: ameta-analysis of observational studies. Ann Rheum Dis. 2017;76(9):1566-74.

82.	Tektonidou MG, Lewandowski LB, Hu J, Dasgupta A, Ward MM. Survival in adults and children with systemic lupus erythematosus: a systematic review and Bayesian meta-analysis of studies from 1950 to 2016. Ann Rheum Dis. 2017;76(12):2009-16.

83.	Tricco AC, Thomas SM, Veroniki AA, Hamid JS, Cogo E, Strifler L, et al. Comparisons of Interventions for Preventing Falls in Older Adults: A Systematic Review and Meta-analysis. JAMA. 2017;318(17):1687-99.

84.	Udell JA, Bonaca MP, Collet J-P, Lincoff AM, Kereiakes DJ, Costa F, et al. Long-term dual antiplatelet therapy for secondary prevention of cardiovascular events in the subgroup of patients with previous myocardial infarction: a collaborative meta-analysis of randomized trials. Eur Heart J. 2016;37(4):390-9.

85.	Valentin N, Camilleri M, Altayar O, Vijayvargiya P, Acosta A, Nelson AD, et al. Biomarkers for bile acid diarrhoea in functional bowel disorder with diarrhoea: a systematic review and meta-analysis. Gut. 2016;65(12):1951-9.

86.	Vancampfort D, Correll CU, Galling B, Probst M, De Hert M, Ward PB, et al. Diabetes mellitus in people with schizophrenia, bipolar disorder and major depressive disorder: a systematic review and large scale meta-analysis. World psychiatry : official journal of the World Psychiatric Association (WPA). 2016;15(2):166-74.

87.	Vancampfort D, Firth J, Schuch FB, Rosenbaum S, Mugisha J, Hallgren M, et al. Sedentary behavior and physical activity levels in people with schizophrenia, bipolar disorder and major depressive disorder: a global systematic review and meta-analysis. World psychiatry : official journal of the World Psychiatric Association (WPA). 2017;16(3):308-15.

88.	Varosy PD, Chen LY, Miller AL, Noseworthy PA, Slotwiner DJ, Thiruganasambandamoorthy V. Pacing as a Treatment for Reflex-Mediated (Vasovagal, Situational, or Carotid Sinus Hypersensitivity) Syncope: A Systematic Review for the 2017 ACC/AHA/HRS Guideline for the Evaluation and Management of Patients With Syncope: A Report of the American College of Cardiology/American Heart Association Task Force on Clinical Practice Guidelines and the Heart Rhythm Society. Circulation. 2017;136(5):e123-e35.

89.	Visrodia K, Singh S, Krishnamoorthi R, Ahlquist DA, Wang KK, Iyer PG, et al. Magnitude of Missed Esophageal Adenocarcinoma After Barrett's Esophagus Diagnosis: A Systematic Review and Meta-analysis. Gastroenterology. 2016;150(3):599-5.

90.	Wang R, Dasgupta A, Ward MM. Comparative efficacy of non-steroidal anti-inflammatory drugs in ankylosing spondylitis: a Bayesian network meta-analysis of clinical trials. Ann Rheum Dis. 2016;75(6):1152-60.

91.	West CP, Dyrbye LN, Erwin PJ, Shanafelt TD. Interventions to prevent and reduce physician burnout: a systematic review and meta-analysis. Lancet (London, England). 2016;388(10057):2272-81.

92.	Willeit P, Welsh P, Evans JDW, Tschiderer L, Boachie C, Jukema JW, et al. High-Sensitivity Cardiac Troponin Concentration and Risk of First-Ever Cardiovascular Outcomes in 154,052 Participants. J Am Coll Cardiol. 2017;70(5):558-68.

93.	Yeo YH, Shiu S-I, Ho HJ, Zou B, Lin J-T, Wu M-S, et al. First-line Helicobacter pylori eradication therapies in countries with high and low clarithromycin resistance: a systematic review and network meta-analysis. Gut. 2018;67(1):20-7.

94.	Younossi Z, Park H, Henry L, Adeyemi A, Stepanova M. Extrahepatic Manifestations of Hepatitis C: A Meta-analysis of Prevalence, Quality of Life, and Economic Burden. Gastroenterology. 2016;150(7):1599-608.

95.	Zaccardi F, Htike ZZ, Webb DR, Khunti K, Davies MJ. Benefits and Harms of Once-Weekly Glucagon-like Peptide-1 Receptor Agonist Treatments: A Systematic Review and Network Meta-analysis. Ann Intern Med. 2016;164(2):102-13.

96.	Zhang X-L, Zhu L, Wei Z-H, Zhu Q-Q, Qiao J-Z, Dai Q, et al. Comparative Efficacy and Safety of Everolimus-Eluting Bioresorbable Scaffold Versus Everolimus-Eluting Metallic Stents: A Systematic Review and Meta-analysis. Ann Intern Med. 2016;164(11):752-63.

97.	Zhang X-L, Zhu Q-Q, Kang L-N, Li X-L, Xu B. Mid- and Long-Term Outcome Comparisons of Everolimus-Eluting Bioresorbable Scaffolds Versus Everolimus-Eluting Metallic Stents: A Systematic Review and Meta-analysis. Ann Intern Med. 2017;167(9):642-54.

98.	Zhao DF, Edelman JJ, Seco M, Bannon PG, Wilson MK, Byrom MJ, et al. Coronary Artery Bypass Grafting With and Without Manipulation of the Ascending Aorta: A Network Meta-Analysis. J Am Coll Cardiol. 2017;69(8):924-36.

99.	Zhao J-G, Zeng X-T, Wang J, Liu L. Association Between Calcium or Vitamin D Supplementation and Fracture Incidence in Community-Dwelling Older Adults: A Systematic Review and Meta-analysis. JAMA. 2017;318(24):2466-82.

100.	Zong G, Gao A, Hu FB, Sun Q. Whole Grain Intake and Mortality From All Causes, Cardiovascular Disease, and Cancer: A Meta-Analysis of Prospective Cohort Studies. Circulation. 2016;133(24):2370-80.
